# Transcriptomic and ChIP-seq Integrative Analysis Reveals Important Roles of Epigenetically Regulated lncRNAs in Placental Development in Meishan Pigs

**DOI:** 10.3390/genes11040397

**Published:** 2020-04-06

**Authors:** Dadong Deng, Xihong Tan, Kun Han, Ruimin Ren, Jianhua Cao, Mei Yu

**Affiliations:** Key Lab of Agricultural Animal Genetics, Breeding and Reproduction of Ministry of Education, College of Animal Science and Technology, Huazhong Agricultural University, Wuhan 430070, China; dadong_deng@163.com (D.D.); xihongTan_920@163.com (X.T.); hankun19910110@163.com (K.H.); ruimin.ren@webmail.hzau.edu.cn (R.R.); jhcao@mail.hzau.edu.cn (J.C.)

**Keywords:** lncRNA, histone modifications, placental development, pig

## Abstract

The development of the placental fold, which increases the maternal–fetal interacting surface area, is of primary importance for the growth of the fetus throughout the whole pregnancy. However, the mechanisms involved remain to be fully elucidated. Increasing evidence has revealed that long non-coding RNAs (lncRNAs) are a new class of RNAs with regulatory functions and could be epigenetically regulated by histone modifications. In this study, 141 lncRNAs (including 73 up-regulated and 68 down-regulated lncRNAs) were identified to be differentially expressed in the placentas of pigs during the establishment and expanding stages of placental fold development. The differentially expressed lncRNAs and genes (DElncRNA-DEgene) co-expression network analysis revealed that these differentially expressed lncRNAs (DElncRNAs) were mainly enriched in pathways of cell adhesion, cytoskeleton organization, epithelial cell differentiation and angiogenesis, indicating that the DElncRNAs are related to the major events that occur during placental fold development. In addition, we integrated the RNA-seq (RNA sequencing) data with the ChIP-seq (chromatin immunoprecipitation sequencing) data of H3K4me3/H3K27ac produced from the placental samples of pigs from the two stages (gestational days 50 and 95). The analysis revealed that the changes in H3K4me3 and/or H3K27ac levels were significantly associated with the changes in the expression levels of 37 DElncRNAs. Furthermore, several H3K4me3/H3K27ac-lncRNAs were characterized to be significantly correlated with genes functionally related to placental development. Thus, this study provides new insights into understanding the mechanisms for the placental development of pigs.

## 1. Introduction

The placenta is an organ that provides nutrients and oxygen to the developing fetus during pregnancy. The pig has an epitheliochorial type of placenta, in which the trophoblast epithelial layer interdigitates with the maternal endometrial epithelial layer to form the folded bilayer [1]. Around gestational day 50 (the gestational length is about 114 days in pigs), the placental fold development reaches a temporary steady state. Thereafter, as gestation progresses, the placental fold increases in depth or complexity to further expand the exchange surface area [2,3,4,5]. Therefore, the placental fold development is a critical contributor to maintaining the function of porcine placentas. In pigs, fetal loss and depression in piglet birth weight are primary factors associated with reproductive performance and were demonstrated to be mainly caused by placental dysfunction [6,7,8,9]. Although many genes and factors have been reported to be involved in the development of the porcine placental fold [3,4,10,11], the mechanism for porcine placental development remains to be fully elucidated.

Long non-coding RNAs (lncRNAs) are known to be larger than 200 nt with little or no coding potential [12,13]. They play a diverse role in biological processes, including transcriptional and post-transcriptional regulation, as well as chromatin remodeling [14,15,16,17,18,19]. In humans, a large number of lncRNAs were identified throughout the genome in term placenta [20]. Several lncRNAs, such as H19 imprinted maternally expressed transcript (H19), X-inactive specific transcript (Xist) and SPRY4 intronic transcript 1 (SPRY4-IT1), were also characterized to be associated with trophoblast development and placental disease [21,22,23,24]. In addition, Bischoff et al. revealed the differences in Xist isoform expression between the commercial swine populations and the Meishan placentas [25]. These findings indicate a significant role of the lncRNA in the regulation of the placental development and function. 

Histone modifications, such as methylation and acetylation, have been demonstrated to be the important epigenetic modifications that have an important impact on gene transcription [26,27,28]. Recent studies revealed that lncRNAs could be epigenetically regulated in a broad range of biological processes, including Alzheimer’s disease (AD), embryonic stem (ES) cells and placental development [22,29,30,31]. Previously, by using ChIP-seq (chromatin immunoprecipitation sequencing) and RNA-seq (RNA sequencing) approaches, we identified the genome-wide profiles of H3K4me3 and H3K27ac in porcine placentas at gestational days 50 (establishment stage of the placental fold) and 95 (expanding stage of the placental fold). In this study, from the perspective of lncRNA genes other than protein-coding genes, we re-analyzed the data sets in an effort to (1) identify the differentially expressed lncRNAs (DElncRNAs) in porcine placentas during the two developmental stages; (2) characterize the DElncRNAs that may be epigenetically regulated; (3) investigate the H3K4me3/H3K27ac–lncRNA–gene pathways that may be associated with placental development in pigs. 

## 2. Materials and Methods

### 2.1. Samples and RNA Preparation

All procedures for the collection of samples were performed according to protocols approved by the biological study animal care and use committee from The Scientific Ethic Committee of Huazhong Agricultural University, Hubei province (HZAUSW-2016-015). Chinese Meishan gilts were raised in the pig farm of Huazhong Agricultural University (Wuhan, China) and were naturally mated with one Meishan boar at the onset of estrus (day = 0) and again 24 h later. The gilts were euthanized on gestational day 50 or 95 (n = 3–5 gilts/gestational day). The placenta (chorioallantoic tissue) was collected and snap-frozen in liquid nitrogen as previously described [11]. The total RNA from each placenta was extracted by using Trizol reagent (Invitrogen, Carlsbad, CA, USA). The quality and quantity of these RNA samples were determined by an Agilent 2100 Bioanalyzer (Agilent Technologies, Santa Clara, CA, USA).

### 2.2. RNA-seq Analysis

In this study, a total of eight libraries (3 and 5 placentas from gestational days 50 and 95, respectively) were constructed for RNA sequencing using an Illumina HiSeq X Ten sequencer (Novogene, Beijing, China). Paired end reads of 2 × 150 base pairs from each sample were generated. Previously, we reported the protein-coding gene expression data based on the RNA-seq data generated from 10 other placental samples of Meishan gilts (6 and 4 placentas from gestational days 50 and 95, respectively) [11]. In the present study, the 2 RNA-seq data sets were combined for further analyses with the aim of identifying the lncRNAs and their co-expressed genes. Thus, the RNA-seq data generated from 18 randomly selected placental samples (9 and 9 placentas from gestational days 50 and 95, respectively) were used in the study and the data were deposited into the NCBI (national center for biotechnology information) Sequence Read Archive database (SRP251127). The reads containing an adapter or poly-N and reads of low quality were removed from the raw reads to generate the clean reads for further analyses. The clean reads were mapped with HISAT2 [32] to the porcine genome sequence (Sscrofa 11.1). The mapped reads of each sample were assembled by both Cufflinks [33] and StringTie [34]. Information about the number and quality of the RNA-seq data is listed in Table S1. Read counts and TPM (transcripts per kilobase of exon model per million mapped reads) for each gene and lncRNA from each sample were calculated using StringTie [34,35]. 

### 2.3. LncRNA Detection

To identify lncRNAs in porcine placenta, we designed a lncRNA analysis pipeline: (1) we compared our porcine placental RNA-seq-assembled transcriptome with Sscrofa 11.1 genome annotation and kept the novel transcripts located in intergenic, intron and anti-sense regions; (2) we removed the transcripts whose length was less than 200 nucleotides or whose number of exons was less than 2 as well as those lowly expressed transcripts (less than 2 individuals with TPM > 1 in the transcript) (3) we assessed the protein-coding potential of the transcripts and distinguished the protein-coding and non-coding transcripts by using coding potential calculator (CPC) [36] and coding potential assessment tool (CPAT) [37]. Transcripts without coding potential were considered to be the candidate lncRNAs.

### 2.4. Differential Expression Analysis

The read counts of each lncRNA and gene were used for differential expression analysis by using edgeR [38]. Genes or lncRNAs with an adjust *p*-value (Benjamini–Hochberg methods) < 0.05 and fold-change >2 were assigned as being differentially expressed. 

### 2.5. lncRNA–Gene Co-Expression Analysis

The Pearson correlation coefficients (PCC) between the lncRNAs and genes were calculated with log2 transformation of the raw TPM. The co-expressed genes of the lncRNAs were identified with the PCC > 0.9 and *p* < 0.0001. 

### 2.6. ChIP-seq Analysis

Previously, we published the ChIP-seq data of H3K4me3 and H3K27ac [11]. The data were generated from 5 porcine placentas (3 and 2 placentas from gestational days 50 and 95, respectively). In this study, we re-analyzed the ChIP-seq data using the same pipeline. Peaks that are within the 3 kb region from the TSS (transcription start sites) of the differentially expressed lncRNAs and that increased or decreased more than 2-fold between gestational days 50 and 95 were defined as the significant differential modifications. 

### 2.7. Gene Ontology (GO) Enrichment Analysis

The co-expressed genes of the differentially expressed lncRNA were used for enrichment analysis. The enrichment of the specific gene ontology (GO) biological processes was analyzed using the DAVID Web-based tool (https://david.ncifcrf.gov). 

### 2.8. Quantitative Real-Time PCR and ChIP-qPCR

Quantitative real-time PCR (qRT-PCR) and ChIP Quantitative PCR (ChIP-qPCR) were carried out for validating the RNA-seq and ChIP-seq data, respectively. Reverse transcription was performed using a PrimeScript RT Reagent Kit with a gDNA Eraser (Takara Bio Inc., Dalian, China). The specific primers of genes and lncRNAs were designed using the software of primer BLAST from NCBI (Table S1). The glyceraldehyde-3-phosphate dehydrogenase (GAPDH) gene was used as a control. The genes and lncRNAs’ expression levels of different samples were compared by the 2^−∆∆CT^ method [39]. Primers for ChIP-qPCR were designed within the significant differentially modified regions determined by the ChIP-seq assay (Table S1). The input DNA sample was used as the negative control. The qRT-PCR and ChIP-qPCR were performed using standard SYBR Premix Ex TaqII (Tli RNaseH Plus; Takara Bio) in a Bio-Rad CFX384 Touch Real-Time PCR Detection System (Bio-Rad Laboratories, Inc., Hercules, CA, USA). The comparison between gestational days 50 and 95 was made using Student’s *t*-test. *p* < 0.05 was considered statistically significant.

## 3. Results

### 3.1. Identification of Differentially Expressed lncRNAs in Porcine Placentas

**Figure 1:**
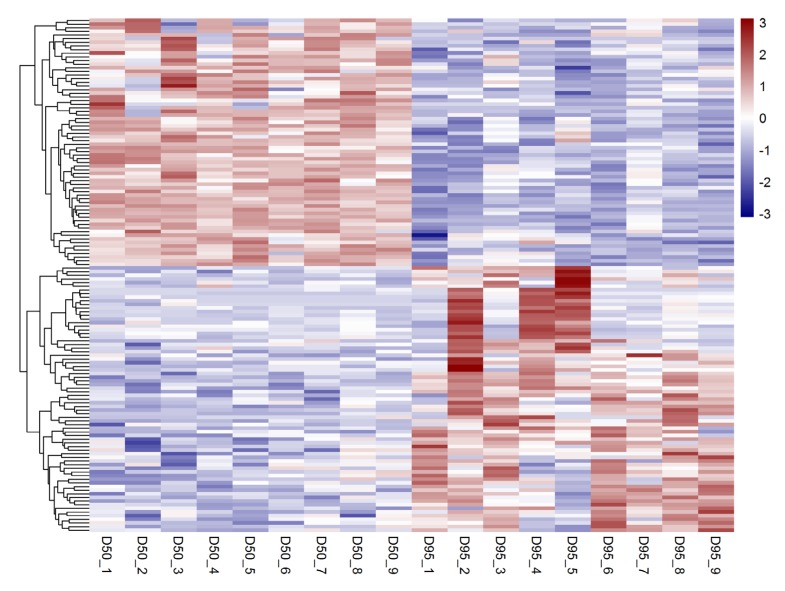
Expression profiles of the differentially expressed long non-coding RNAs (lncRNAs) in the porcine placentas on gestational days 50 and 95 by RNA-seq (adjusted *p*-value < 0.05). D50, gestational day 50; D95, gestational day 95.

In total, 690 putative lncRNAs were identified to be expressed in porcine placentas (chorioallantoic tissues) on gestational days 50 and 95. Among these, 479 lncRNAs were transcribed from intergenic regions, 206 lncRNAs were transcribed as overlapping with and in the antisense orientation to a reference and five lncRNAs were transcripts falling entirely within a reference intron (Table S2). In addition, a total of 141 lncRNAs were found to be differentially expressed in placentas between gestational days 50 and 95 (adjusted *p*-value < 0.05; fold change > 2), including 73 up-regulated and 68 down-regulated lncRNAs on gestational days 95 (Figure 1 and Table S3).

### 3.2. Analysis of the Potential Function of the Differentially Expressed LncRNAs

**Figure 2:**
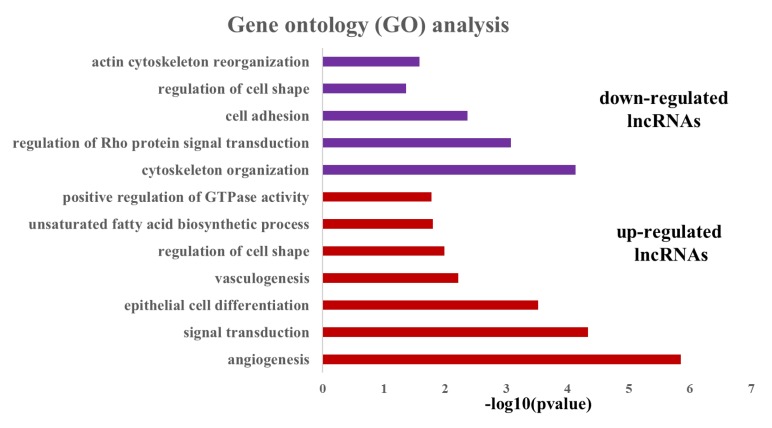
Gene ontology analysis of the co-expressed genes with the differentially expressed lncRNAs (DElncRNAs).

To understand the potential function of the differentially expressed lncRNAs (DElncRNAs), the differentially expressed genes (DEgenes) were detected using the same data set generated from the 18 porcine placental samples. A total of 2208 genes were found to be differentially expressed between gestational days 50 and 95 (including 1689 up-regulated and 519 down-regulated genes, adjusted *p*-value < 0.05, fold change > 2, Table S3). Then, we investigated the co-expression relationships between the DElncRNAs and DEgenes using Pearson’s correlation analysis. In total, 1002 significantly co-expressed lncRNA–gene pairs (*p* < 0.0001, PCC^2^ > 0.8) were identified, in which 72 DElncRNAs (including 37 up-regulated and 35 down-regulated lncRNAs, respectively) and 569 DEgenes were involved (Table S4). Subsequently, gene ontology (GO) analysis was conducted for the 569 co-expressed DEgenes by using DAVID online tools (Figure 2 and Table S5). The most significantly overrepresented terms for the co-expressed DEgenes of the down-regulated lncRNAs were involved in cytoskeleton organization, the regulation of Rho protein signal transduction, cell adhesion, actin cytoskeleton reorganization and the regulation of cell shape, whereas the most significantly overrepresented terms for the co-expressed DEgenes of the up-regulated lncRNAs were related to angiogenesis, signal transduction, epithelial cell differentiation, vasculogenesis, regulation of cell shape, the unsaturated fatty acid biosynthetic process and the positive regulation of GTPase activity.

### 3.3. Investigation of the Role of H3K4me3 and H3K27me3 Modifications in Regulation of the lncRNA Expression in Porcine Placentas

**Figure 3:**
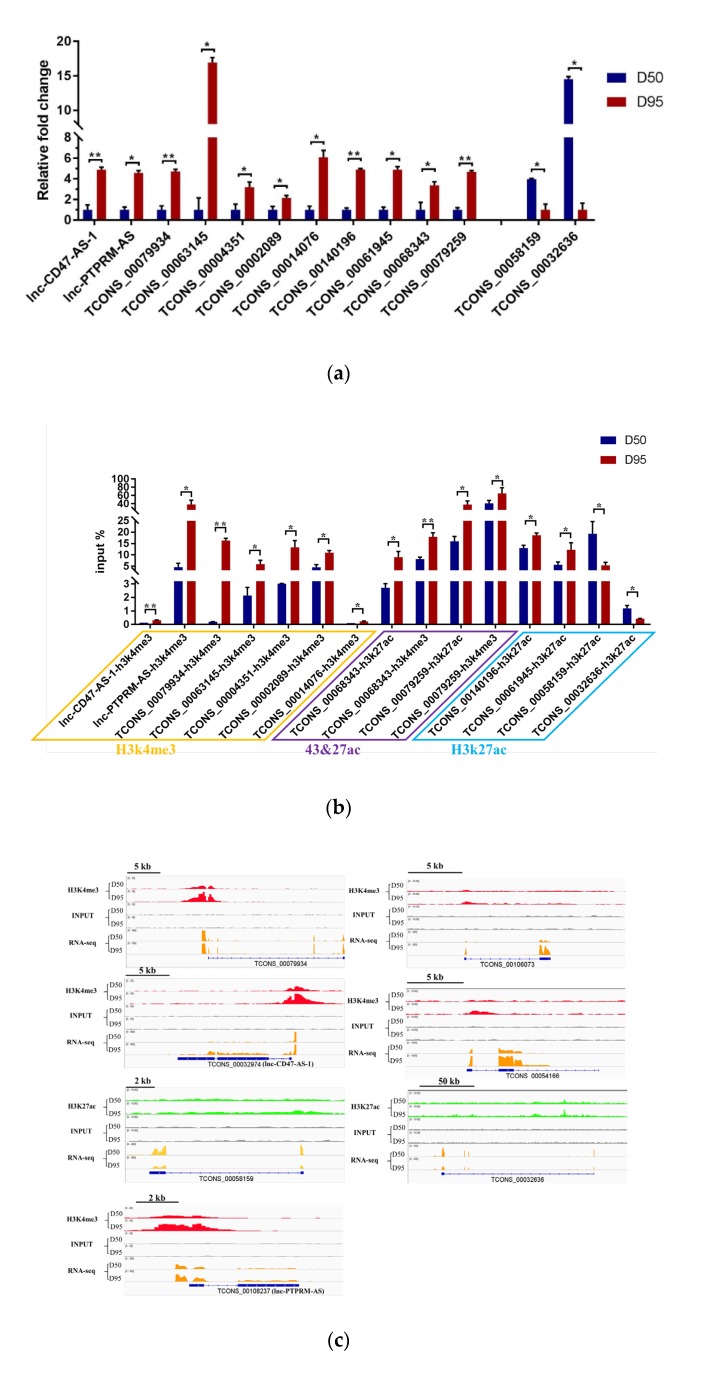

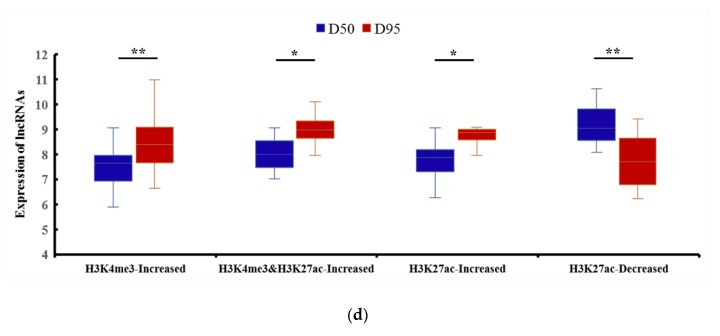
DElncRNAs were regulated by the H3K4me3 and H3K27ac modifications. (**A**) Quantitative real-time PCR (qRT-PCR) validation of the differentially expressed lncRNAs in porcine placenta on gestational days 50 and 95. (**B**) ChIP Quantitative PCR (ChIP-qPCR) validation of the H3K4me3 and H3K27ac modifications of the differentially expressed lncRNAs in porcine placenta on gestational days 50 and 95. (**C**) IGV (Integrative Genome Viewer) views of the H3K4me3 and/or H3K27ac modification patterns. (**D**) Box plots of the expression value of the lncRNAs that were modified by H3K4me3 and/or H3K27ac. Data are represented as mean + SEM, n = 3. * *p* < 0.05; ** *p* < 0.01. D50, gestational day 50; D95, gestational day 95.

Previously, we reported the genome-wide maps of H3K4me3 and H3K27ac in placentas from gestational days 50 and 95 in pigs [11]. In the current study, the data were re-analyzed to identify those DElncRNAs that may be epigenetically regulated by the H3K4me3 and H3K27ac modifications. The significant differential modifications between the two stages within the 3 kb region from the TSS of the 37 DElncRNAs were detected, including 32 H3K4me3 (increased signals: 32; decreased signals: 0) and 16 H3K27ac (increased signals: 7; decreased signals: 9) peaks (Table S6), respectively. Of these 37 DElncRNAs, four DElncRNAs had both H3K4me3- and H3K27ac-increased regions, 22 DElncRNAs had H3K4me3-increased regions, three DElncRNAs had H3K27ac-increased regions and eight DElncRNAs had H3K27ac-decreased regions (Table S6). We next confirmed that the expression levels of 13 DElncRNAs and the enrichments of H3K4me3 and/or H3K27ac in the 13 DElncRNAs (including seven lncRNAs enriched with H3k4me3, four lncRNAs enriched with H3k27ac and two lncRNAs enriched with both H3K4me3 and H3K27ac modifications, respectively) were consistent with the results from the RNA-seq and ChIP-seq data (Figure 3A-C), respectively. Furthermore, the expression levels of the 37 DElncRNAs were quantified by using the RNA-seq data: the results showed that the increased levels of the 29 up-regulated lncRNAs were significantly associated with the increased enrichments of H3K4me3 and/or H3K27ac, while the decreased levels of the eight down-regulated lncRNAs were significantly associated with the decreased enrichment of H3K27ac (Figure 3D).

### 3.4. Validation of the Expression Pattern of DEgenes Co-Expressed with the H3K4me3- and/or H3K27ac- Modified DElncRNAs 

**Figure 4:**
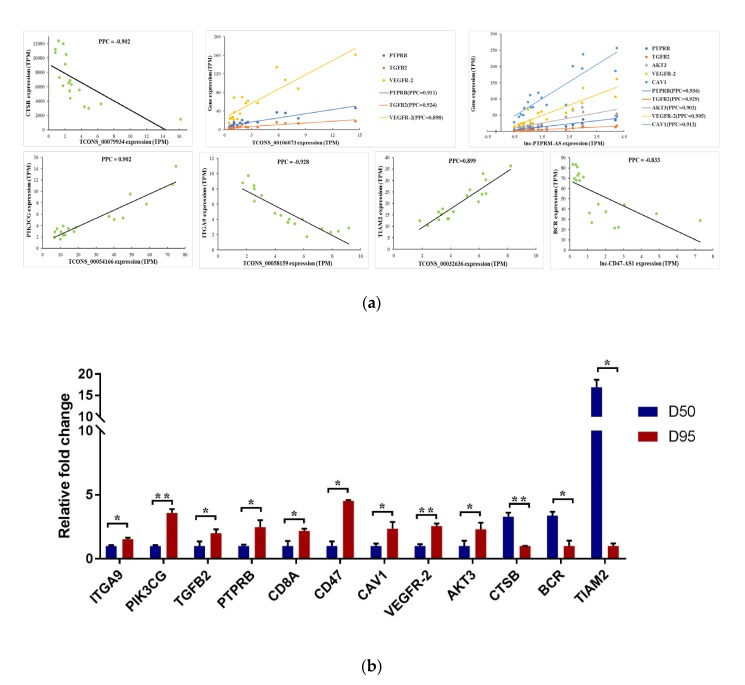
Validation of the expression pattern of the DEgenes co-expressed with the H3K4me3- and/or H3K27ac-modified DElncRNAs. (**A**). A scatterplot of the DElncRNAs and co-expression genes’ expression levels in individual placental samples determined by RNA-Seq. (**B**). Quantitative RT-PCR validation of the DEgenes co-expressed with the DElncRNAs. PCC, Pearson correlation coefficient; data are represented as mean + SEM, n = 3. * *p* < 0.05; * *p* < 0.01.

Of the 13 DElncRNAs whose expression levels were confirmed to be significantly associated with the enrichments of H3K4me3 and/or H3K27ac in placentas during gestational days 50 and 95 in pigs, seven DElncRNAs (lncRNAs *TCONS_00079934*, *TCONS_00106073*, *lnc-PTPRM-AS*, *TCONS_00054166*, *lnc-CD47-AS-1*, *TCONS_00058159* and *TCONS_00032636*) were found to have a strong co-expression relationship with 12 genes (Figure 4A). Then, the expression pattern of the genes co-expressed with these DElncRNAs was validated using qRT-PCR. As shown in Figure 4B, nine genes were confirmed to be up-regulated on gestational day 95, namely *ITGA9* (integrin subunit alpha 9), *PIK3CG* (phosphatidylinositol 4,5-bisphosphate 3-kinase catalytic), *TGFB2* (transforming growth factor beta 2)*, PTPRB* (protein tyrosine phosphatase, receptor type B)*, CD8A* (T-cell surface glycoprotein CD8 alpha chain)*, CD47* (leukocyte surface antigen CD47)*, CAV1* (caveolin 1)*, VEGFR-2* (kinase insert domain receptor)*,* and *AKT3* (AKT serine/threonine kinase 3), while three genes were validated to be down-regulated on gestational day 95, namely *CTSB* (cathepsin B)*, BCR* (BCR activator of RhoGEF and GTPase)*,* and *TIAM2* (T-cell lymphoma invasion and metastasis 2). 

## 4. Discussion

In this study, the lncRNAs that were differentially expressed (DElncRNAs) in porcine placentas from the two crucial placental fold developmental stages were detected. The DEgenes co-expressed with these DElncRNAs were found to be enriched in the pathways related to angiogenesis, cell adhesion, cytoskeleton organization and epithelial cell differentiation. In addition, the DElncRNAs that were marked by H3K4me3 and/or H3K27ac were identified and the expression of these DElncRNAs was detected to be significantly correlated to the changes in the level of H3K4me3 and/or H3K27ac. Furthermore, the H3K4me3/H3K27ac–lncRNA–gene pathways were validated in the placentas of pigs from the two crucial placental fold developmental stages.

As gestation progresses in pigs, the demand of the fetuses for nutrient uptake increases rapidly, thus the placental fold develops further for increasing the maternal–fetal exchange surface area and expanding the maternal and fetal vascular network [2,4,7,40]. A number of genes and miRNAs, such as heparanase (HPSE), hypoxia inducible factor 1a (HIF1A), vascular endothelial growth factor A (VEGFA) and miR-29a, were reported to be functionally related to placental development [3,10,41,42,43]. Recently, RNA-seq data analysis was performed on the placentas of Landrace × Yorkshire gilts on gestational days 60 and 90, as well as the farrowing day. The mRNAs and lncRNAs that were responsible for the bile acid metabolism in placentas were identified [44]. We reported here the lncRNAs that were differentially expressed in the placentas (chorioallantoic tissues) of Meishan gilts from the two crucial placental fold developmental stages: gestational days 50 (establishment stage of placental fold) and 95 (expanding stage of placental fold) and the differential expression of the DElncRNAs was validated by using qRT-PCR. Our finding is in agreement with the previous report demonstrating the role of lncRNAs in regulating the gene expression in the placentas of pigs [44]. On the other hand, in addition to vasculature development, it has been documented that the placental trophoblast cells undergo differentiation and change in cell shape during the development of the placental fold in pigs [3,45,46,47,48]. Our DElncRNA-DEgene co-expression network analysis revealed that these DElncRNAs were mainly enriched in pathways of cell adhesion, cytoskeleton organization, epithelial cell differentiation and angiogenesis, indicating that the DElncRNAs are related to the major events that occur during the placental fold development [4,11,45,49,50]. These findings suggest the important role of these DElncRNAs in the development of the placental fold. 

There is growing evidence that DNA methylation and histone modification can impact lncRNA expression by altering the chromatin structure [29,51,52,53]. Therefore, we performed an integrative analysis of the lncRNA data and our previously reported ChIP-seq data of H3K4me3 and H3K27ac modifications derived from the placentas of pigs [11]. A total of 37 DElncRNAs (including 29 up-regulated lncRNAs and 8 down-regulated lncRNAs on gestational day 95) were identified to contain H3K4me3 and/or H3K27ac marks within a 3 kb region from the TSS. H3K4me3 and H3K27ac are two of the active chromatin marks that have been illustrated to be associated with active transcription [54,55,56,57,58]. As expected, we found that those up-regulated lncRNAs were associated with the increased levels of H3K4me3 and/or H3K27ac, while those down-regulated lncRNAs were associated with the decreased H3K27ac levels. The findings indicate that the expression of the lncRNAs in the placentas of pigs were at least in part epigenetically regulated by the two histone modifications. Furthermore, we validated that five lncRNAs with increased H3K4me3 and upregulation in expression were significantly co-expressed with 10 DEgenes including *CTSB*, *CD8A*, *PTPRB*, *TGFB2*, *VEGFR-2*, *AKT3*, *CAV1*, *PIK3CG*, *BCR* and *CD47*, while two lncRNAs with decreased H3K27ac and downregulation in expression were significantly co-expressed with two DEgenes: *ITGA9* and *TIAM2*. *CTSB* is a lysosomal cysteine protease that can degrade the extracellular matrix (ECM) and was confirmed to be expressed in the chorionic epithelium of the placenta in pigs [59,60]. *CD8A* encodes the alpha chain of CD8, which is a co-receptor for the MHC class I molecule peptide complex and plays an essential role in the immune response [61]. Five of the DEgenes, *PTPRB, TGFB2, AKT3, PIK3CG* and *ITGA9*, are involved in a wide variety of biological processes such as epithelium morphogenesis, angiogenesis and immune homeostasis [62,63,64,65,66]. *VEGFR-2* is one of the VEGF transmembrane tyrosine kinase receptors. It has been reported that *VEGFR-2* functions in the regulation of VEGF by interaction with *CD47*, thus the two genes play key roles in angiogenesis and vascular permeability [67,68,69,70]. The integral membrane protein encoded by *CAV1* is the main component of the caveolar membranes and acts with diverse functions in cells, such as cell differentiation and movement [71,72,73]. *BCR* plays a role in regulating small GTP-binding proteins, such as *CDC42* (cell division cycle 42). *CDC42* was detected to be expressed in the placenta of pigs and functions in epithelial tissue morphogenesis [74,75]. *TIAM2* has been characterized to promote the cell proliferation and invasion [76,77]. 

**Figure 5:**
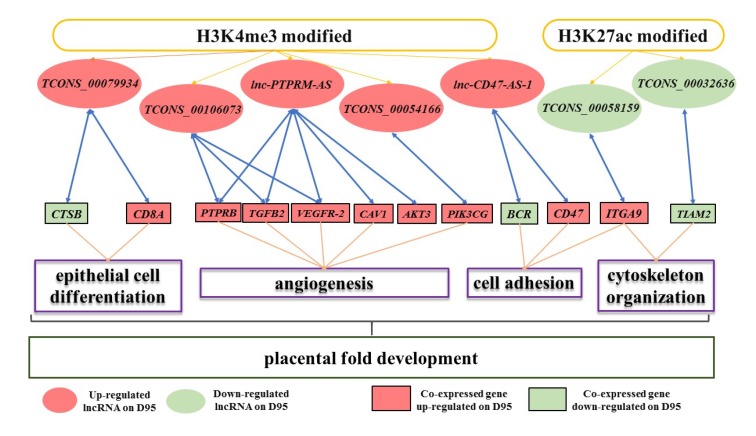
Schematic illustration of the potential roles of H3K4me3/H3K27ac–lncRNA–gene pathways on porcine placental development.

Overall, we validated that changes in the expression levels of seven lncRNAs were significantly associated with changes in H3K4me3 and/or H3K27ac levels. In addition, 12 genes which play roles in epithelial cell differentiation, angiogenesis, cell adhesion and cytoskeleton organization were validated to be co-expressed with the seven lncRNAs during the placental development of pigs. Of these lncRNAs, *lnc-CD47-AS-1* is overlapped with *CD47* on the opposite stand and *Lnc*-*TCONS_00079934* is localized near the *CD8A* gene. These two lncRNAs may regulate the two genes by recruiting regulatory complexes through RNA–protein interactions [18,78,79]. In addition, five lncRNAs (*TCONS_00079934*, *TCONS_00106073*, *TCONS_00054166*, *TCONS_00058159* and *TCONS_00032636*) are intergenic lncRNAs and *lnc-PTPRM-AS* is an antisense lncRNA (Table S6). These six lncRNAs and their corresponding co-expressed genes are located on the different chromosomes, suggesting that these lncRNAs may regulate gene expression in trans through the mechanism of proximity transfer [79,80,81]. Thus, we speculated that the epigenetic transcriptional marks, H3K4me3 and H3K27ac, may have an impact on the expression of the lncRNAs and these lncRNAs may act in cis or in trans to affect the transcription of the co-expressed genes during the placental development of pigs (Figure 5).

In conclusion, this study identified the lncRNAs that are differentially expressed in the placentas of pigs obtained from two crucial placental fold developmental stages. In addition, we characterized DElncRNAs whose expression changes are correlated with altered levels of H3K4me3 and/or H3K27ac. Furthermore, H3K4me3/H3K27ac–lncRNA–gene pathways are suggested to play important roles in the placental fold development of pigs. 

## Supplementary Materials

The following are available online at https://www.mdpi.com/2073-4425/11/4/397/s1, Table S1: Information of the RNA-Seq data and primers, Table S2: Information of the identified lncRNAs, Table S3: The differentially expressed lncRNAs and genes, Table S4: The significantly co-expressed lncRNA-gene pairs, Table S5: Gene ontology analysis data, Table S6: Information of H3K4me3/H3K27ac-lncRNAs.
