# Supplementary material for: Transcriptomic and ChIP-seq Integrative Analysis Reveals Important Roles of Epigenetically Regulated lncRNAs in Placental Development in Meishan Pigs
Source: Genes (Basel). 2020 Apr 6;11(4):397. doi: 10.3390/genes11040397 (PMC7230623; doi:10.3390/genes11040397)
Supplement: Supplementary file 1 [file genes-11-00397-s001.zip › Supplementary Table 1.doc]

Supplementary Table 1-1. RNA-Seq data quality

| Sample | Total reads | Type | length of reads | Mapping ratio (%) | Mapped reads |
| --- | --- | --- | --- | --- | --- |
| 110426M50L1 | 51854436 | Paired-end | 150bp | 72.20% | 37438903 |
| 110504M50L1 | 35889588 | Paired-end | 150bp | 71.70% | 25732835 |
| 151211M50R2 | 40052132 | Paired-end | 150bp | 69.10% | 27676023 |
| 110222M95L2 | 33185286 | Paired-end | 150bp | 68.70% | 22798291 |
| 160507M95L2 | 37189148 | Paired-end | 150bp | 58.00% | 21569706 |
| 160828M95R1 | 46500106 | Paired-end | 150bp | 68.00% | 31620072 |
| 160925M95L1 | 34436626 | Paired-end | 150bp | 75.40% | 25965216 |
| 160925M95L2 | 34041124 | Paired-end | 150bp | 78.50% | 26722282 |
| ^*^170418M50L1 | 64740342 | Paired-end | 150bp | 95.00% | 61501032 |
| ^*^170418M50R1 | 61255666 | Paired-end | 150bp | 95.04% | 58219223 |
| ^*^170422M50L1 | 74865306 | Paired-end | 150bp | 95.40% | 71423416 |
| ^*^170422M50R2 | 69471338 | Paired-end | 150bp | 95.24% | 66167734 |
| ^*^170427M50L1 | 80505654 | Paired-end | 150bp | 95.38% | 76790098 |
| ^*^170427M50R1 | 81386592 | Paired-end | 150bp | 95.32% | 77581272 |
| ^*^170522M95L1 | 68174039 | Paired-end | 150bp | 95.21% | 64911842 |
| ^*^170522M95L3 | 76109667 | Paired-end | 150bp | 95.41% | 72613334 |
| ^*^170522M95R2 | 64737065 | Paired-end | 150bp | 95.14% | 61588579 |
| ^*^170625M95R1 | 70088764 | Paired-end | 150bp | 95.06% | 66627602 |

^*^ Data was published previously by Han et al. (2019).

Supplementary Table 1-2. List of oligonucleotide sequences used in this study.

**qRT-PCR Primers**

| Gene and LncRNA | Forward Primer (5'-3') | Reverse Primer (5'-3') |
| --- | --- | --- |
| *ITGA9* | CCGGGAAGACCGAGATGATG | ATGTAACAGAAGCCGTGGGG |
| *PIK3CG* | ACCTCCGCTGCTTTATCAGG | GCTGGTGGGCAAAACAAACT |
| *TGFB2* | GCGCTACATCGACAGCAAAG | TGCAGCAGGGACAGTGTAAG |
| *PTPRB* | CGTTAAATCTGCGCTCTGCC | TTCCTTCTCGTGGCAAGTCC |
| *CD8A* | GGAATAAGACAGCCGAGGGG | GGCTTTGCTGGCAAGAAGAC |
| *CD47* | TTGATGGCGCTCAGCACATA | ACGGCATCACGCTTATCCAT |
| *CAV1* | TCCCAAGCATCTCAACGACG | ATGCCAAAGAGGGCAGACAG |
| *VEGFR-2* | ATGAAGGCCTGTACACCTGC | ACGATGACAAGAAGCAGCCA |
| *AKT3* | ACCGCACACGTTTCTATGGT | AGATTCTCCAACTCTGGTGCC |
| *CTSB* | CCATCTCTGACCGGATCTGC | GAGGGAAAGCCACCGTTACA |
| *BCR* | AGAGGTCCAAGGTGCCCTAT | GCGTTCACGTCCATCTCACT |
| *TIAM2* | GACGACTACCATCAGGCTCG | CTTGGTGCTGTTGGCTTTCC |
| *lnc-CD47-AS-1* | CTATACGCCTCTCGCCTCTG | GCTCCTCTGCAGCTCATTTC |
| *lnc-PTPRM-AS* | GCTCTGCGGCTTCATTGC | TTCCATTTGGGCTCAAGGCT |
| *TCONS_00079934* | GTCGCAGACACAGCTCAGAT | GGATGGACTGGGAGTTTGGG |
| *TCONS_00063145* | GCCACTCTGAGCATGGACTT | ACCACAATCCTTCACGGTCC |
| *TCONS_00004351* | GGTGTCCTCTCCTTCCACGG | GGCTCTTTCTGGGCTTTGAGT |
| *TCONS_00002089* | GGCCTCACCAGCGACATATT | TGCAGAATTGTGGGGGTCTC |
| *TCONS_00014076* | GGAACTCGAGAGCAGAGCTA | ACACCTCTTGATCCGGTGGA |
| *TCONS_00068343* | AGTCACTGGCACATACCGATG | TGAGGACGATGACCCAGACG |
| *TCONS_00079259* | TTGCTCTAGCCCCCAAGGAC | GATATCTTGGCGCCGGATGG |
| *TCONS_00140196* | TGGACGGCAGGTGGAAATAG | ACGGCCTTTGATGTGGTGAT |
| *TCONS_00061945* | GCTCTGTGGCTGCTTTCAGA | CCTGGGGACTGTAGGGTGATA |
| *TCONS_00058159* | AAGGTTTGTCGCCCTTTTTGG | GTGTCCAGGAGCCTGTAGTT |
| *TCONS_00032636* | GGCTGAGTTCACTTTCCATGTG | AAACCAAGTTCCTACCCGCA |

**ChIP-qPCR Primers**

|  | Forward Primer (5'-3') | Reverse Primer (5'-3') |
| --- | --- | --- |
| *lnc-CD47-AS-1-h3k4me3* | ACTTCAAACGGAGCACCTCG | GCGCACTTTCATCGCAAAAG |
| *lnc-PTPRM-AS-h3k4me3* | GAGCAGCTGTAGACGCTAGG | CAGGTGAGAGCAGCACAGAA |
| *TCONS_00079934-h3k4me3* | GCCACAAACCTTGACCAACC | GGTGGTTTCCTGGTGTCCTT |
| *TCONS_00063145-h3k4me3* | GCGCTTAGACGTCCCTTCTC | GCTCCCTCGCACTGATACAA |
| *TCONS_00004351-h3k4me3* | AGGAAGAAAGGTGAGCGTGG | GACCAATTTCACAGCTGGCG |
| *TCONS_00002089-h3k4me3* | GGCCTCACCAGCGACATATT | CAAAACCGAGCCCGAATCAC |
| *TCONS_00014076-h3k4me3* | CTGCCTGGCGTGAGAGTTTA | AGCATCGCCTCGAGATAAGC |
| *TCONS_00068343-h3k27ac* | CAGCGGGTTATCTGTCCAACT | CTGCGCAGATGCCTTTTTCC |
| *TCONS_00068343-h3k4me3* | CAGCGGGTTATCTGTCCAACT | CTGCGCAGATGCCTTTTTCC |
| *TCONS_00079259-h3k27ac* | CCAGCGCCAAGAGCTTATGA | AACATCTGTGGGTTTCCGCT |
| *TCONS_00079259-h3k4me3* | TCTATGCTGGGGACACGAGA | AGCCTCGCTTAGCTCTTTGG |
| *TCONS_00140196-h3k27ac* | TATTTCCACCTGCCGTCCAG | ATGGGGCTAGAACGTGGTTG |
| *TCONS_00061945-h3k27ac* | CAGCTGTGGCCTATGGAAGT | GTGGGTTAAGGATCTGGCGT |
| *TCONS_00058159-h3k27ac* | CAGCAAGGGACATGTCTCAGAA | TCCTGCCTCTGCCTGAAGAT |
| *TCONS_00032636-h3k27ac* | TGGAAGGAGGCGGCAAATAG | CTTTCGAGAGAGGTCTGGGC |
